# Measuring size and composition of species pools: a comparison of dark diversity estimates

**DOI:** 10.1002/ece3.2169

**Published:** 2016-05-20

**Authors:** Francesco de Bello, Pavel Fibich, David Zelený, Martin Kopecký, Ondřej Mudrák, Milan Chytrý, Petr Pyšek, Jan Wild, Dana Michalcová, Jiří Sádlo, Petr Šmilauer, Jan Lepš, Meelis Pärtel

**Affiliations:** ^1^ Institute of BotanyThe Czech Academy of SciencesDukelská 135CZ‐379 82TřeboňCzech Republic; ^2^ Department of BotanyUniversity of South BohemiaNa Zlaté stoce 1CZ‐370 05České BudějoviceCzech Republic; ^3^ Department of Botany and ZoologyMasaryk UniversityKotlářská 2CZ‐611 37BrnoCzech Republic; ^4^ Institute of Ecology and Evolutionary BiologyNational Taiwan UniversityRoosevelt Rd. 110617TaipeiTaiwan; ^5^ Institute of BotanyThe Czech Academy of SciencesLidická 25/27CZ‐602 00BrnoCzech Republic; ^6^ Department of Forest EcologyFaculty of Forestry and Wood SciencesCzech University of Life Sciences PragueKamýcká 129CZ‐165 21Prague 6 – SuchdolCzech Republic; ^7^ Institute of BotanyThe Czech Academy of SciencesZámek 1CZ‐252 43PrůhoniceCzech Republic; ^8^ Department of EcologyFaculty of ScienceCharles University in PragueViničná 7CZ‐128 44PragueCzech Republic; ^9^ Department of Botany and ZoologyCentre for Invasion BiologyStellenbosch UniversityMatieland7602South Africa; ^10^ Faculty of Environmental SciencesCzech University of Life Sciences PragueKamýcká 129CZ‐165 21Prague 6 – SuchdolCzech Republic; ^11^ Department of Ecosystem BiologyFaculty of ScienceUniversity of South BohemiaBranišovská 1970CZ‐370 05České BudějoviceCzech Republic; ^12^ Biology CentreThe Czech Academy of SciencesBranišovská 31370 05České BudějoviceCzech Republic; ^13^ Institute of Ecology and Earth SciencesUniversity of TartuLai 4051005TartuEstonia

**Keywords:** Beals smoothing, biodiversity monitoring, Biomod, dark diversity, Ellenberg indicator values, method comparison, species distribution modeling

## Abstract

Ecological theory and biodiversity conservation have traditionally relied on the number of species recorded at a site, but it is agreed that site richness represents only a portion of the species that can inhabit particular ecological conditions, that is, the habitat‐specific species pool. Knowledge of the species pool at different sites enables meaningful comparisons of biodiversity and provides insights into processes of biodiversity formation. Empirical studies, however, are limited due to conceptual and methodological difficulties in determining both the size and composition of the absent part of species pools, the so‐called dark diversity. We used >50,000 vegetation plots from 18 types of habitats throughout the Czech Republic, most of which served as a training dataset and 1083 as a subset of test sites. These data were used to compare predicted results from three quantitative methods with those of previously published expert estimates based on species habitat preferences: (1) species co‐occurrence based on Beals' smoothing approach; (2) species ecological requirements, with envelopes around community mean Ellenberg values; and (3) species distribution models, using species environmental niches modeled by Biomod software. Dark diversity estimates were compared at both plot and habitat levels, and each method was applied in different configurations. While there were some differences in the results obtained by different methods, particularly at the plot level, there was a clear convergence, especially at the habitat level. The better convergence at the habitat level reflects less variation in local environmental conditions, whereas variation at the plot level is an effect of each particular method. The co‐occurrence agreed closest the expert estimate, followed by the method based on species ecological requirements. We conclude that several analytical methods can estimate species pools of given habitats. However, the strengths and weaknesses of different methods need attention, especially when dark diversity is estimated at the plot level.

## Introduction

Describing and understanding the patterns of species diversity presents a major challenge for both theoretical ecologists and conservationists (Gotelli and Colwell 2001; Carstensen et al. 2013; Reese et al. 2014; Lewis et al. 2016a). Ecological theory and biodiversity conservation have traditionally relied on the number of species recorded at a site, that is, species richness, measured using different sampling techniques and monitoring schemes (Bruun 2000; de Bello et al. 2010; Pärtel et al. 2011). However, this measure of diversity is only a portion of the “habitat‐specific species pool” of a site, that is, all the species in a region that can potentially inhabit the ecological conditions at that site (Eriksson 1993; Cornell and Harrison 2014; Zobel 2016). Some species remain undetected because of incomplete biodiversity monitoring, both in space and time, due to limited effort or resources. Some sites, however, might lack otherwise suitable species from the surrounding region due to the isolation of the site or the poor dispersal ability of these species (Riibak et al. 2015). In addition, the biotic and abiotic conditions at a site might not temporarily allow some species to establish or cause temporary local extinctions (de Bello et al. 2012; Carstensen et al. 2013; Lessard et al. 2016).

Considering only the recorded diversity can have important drawbacks for ecological theory and biodiversity conservation (Eriksson 1993; Pärtel et al. 2011; Karger et al. 2016; Lewis et al. 2016a; Zobel 2016). For example, the absolute values of species richness, particularly when estimated at one spatial scale, are of a limited value for comparing biodiversity across ecosystems, regions, or taxonomic groups if not relativized to its potential values (Gotelli and Colwell 2001; Pärtel et al. 2011). Also, understanding the mechanisms regulating biodiversity in assemblages at a particular site requires identifying, which and how many species have been excluded during the assembling process (de Bello et al. 2012; Cornell and Harrison 2014), thereby highlighting the processes causing community saturation (Szava‐Kovats et al. 2013). Finally, the absence of species can reflect dispersal limitations and local extinctions, which are of concern to nature conservationists, but also relevant from a restoration and invasion point of view (Pärtel et al. 2011; Kalusová et al. 2014).

Despite the ecological importance of species pools, empirical studies have been limited due to conceptual and methodological difficulties in determining both their size and composition (Karger et al. 2016). The term “species pool” is used with different meanings in the literature (Cornell and Harrison 2014; Zobel 2016). Sometimes it includes all the species present in a particular area without regard to the specific ecological conditions at the target site. This is generally easy to measure when regional lists of flora/fauna or species occurrence maps are available. Here we refer to the “habitat‐specific species pool” (hereafter called “species pool” for simplicity), which includes all the species in a region that can inhabit the ecological conditions at a target site and defines species pools in terms of species habitat preferences. The species that are not recorded at a target site, but belong to its species pool, constitute the “dark diversity” of that site (Pärtel et al. 2011), which like the dark matter in the universe is known to exist but is not visually observable. Different techniques can potentially provide estimates of the dark diversity. Exploring the number of species that can potentially occupy a site is not uncommon in ecology (Bruun 2000; Dupré 2000; Gotelli and Colwell 2001; Ozinga et al. 2005; Carstensen et al. 2013; Cornell and Harrison 2014; Lewis et al. 2016b), as the absence of a species might be as scientifically interesting as its presence. However, there are now several methods for estimating, among other things, the size of species pools (Gotelli and Colwell 2001; Shtilerman et al. 2014), but fewer methods for estimating both their size and composition. Eriksson (1993) suggested that it was necessary to consider the species pool when studying the effect of regional processes on local diversity patterns, but recognized that one of the major difficulties in doing this is the accuracy of the estimates of species pools. Recently, there has been a marked increase in interest in determining realistic estimates of species pools based on repeatable and transparent analytical approaches (Carstensen et al. 2013; Cornell and Harrison 2014; Lewis et al. 2016b; Zobel 2016). However, this toolbox is still being developed.

One way to estimate both the size and composition of the species pool for a site is through extensive sampling of habitat types within a region; this is rather time consuming if many communities are considered, also it is difficult to find all the potential species (Sádlo et al. 2007). Information provided by local experts, based on extensive field experience, is arguably a good source of information but is rarely available. Alternatively, one can consider using one of the various computational approaches. Dupré (2000) shows that species pool estimates based on extensive field sampling are similar to those based on expert knowledge of the different types of vegetation and the species inhabiting them. Dupré (2000) also considers an approach based on species ecological requirements, developed by Pärtel et al. (1996). In this approach, the characteristics of the environment and the ecological responses of species are used to filter out the species of a regional flora based on their known environmental requirements and define the size of the species pool for different communities. This approach is based on the use of Ellenberg indicator values (Ellenberg et al. 1992), which indicate ecological preferences of plant species (i.e., realized niche) along environmental gradients. Ewald (2002) proposes another method based on the likelihood of species co‐occurring. This approach is based on the idea that if some species are frequently found together, the presence of some of them at a site would indicate that both the biotic and abiotic conditions at that site are suitable for the other species. This approach requires large sets of vegetation plots and is generally applied using the index of sociological favourability (Beals 1984), which is also called Beals smoothing. Other approaches are used to predict species potential composition from environmental conditions at target sites (Ozinga et al. 2005). Recently, for example, various species distribution modeling techniques (Guisan and Thuiller 2005) have been successfully used to determine the potential range of environmental conditions suitable for a species (Parolo et al. 2008; Karger et al. 2016). Ecological habitat characteristics and species occurrence data are becoming increasingly available for many locations in several open repositories (e.g., http://www.gbif.org/species). The level of information on habitats and land cover units is also increasing at both the country level and larger scales (Chytrý et al. 2016).

The above set of techniques are potential tools for estimating species pools, particularly the part formed by absent species (dark diversity). Each method requires a different type of data and is based on a different mathematical approach (Table 1). A thorough comparison of available methods can shed light on whether, and to what extent, dark diversity can be accurately estimated. In fact, despite the availability of different techniques for estimating species pools, there are no systematic comparisons of the results obtained using these methods for large regions and different types of vegetation. Recently, Lewis et al. (2016b) showed how the Beals and Ellenberg approaches could be used to predict additional species that would be recorded immediately around the plots sampled within a particular type of vegetation and thus recording part of the potential species for a site. Here, we compare techniques that are used to estimate the dark diversity for different types of vegetation (habitats). We used a large dataset of community samples (>50,000 vegetation plot records from the Czech National Phytosociological Database; Chytrý and Rafajová 2003) and three analytical methods (Table 1 and Methods). We compared the results obtained using these methods and those results with the best available expert evaluations, based on types of vegetation in the region studied (Sádlo et al. 2007).

**Table 1:** Summary of the analytical approaches used to estimate the habitat‐specific species pool at a site (see the main text for more details)

Method	Description	Data type	Thresholds	Observations
Species co‐occurrence patterns (e.g., Beals smoothing)	Based on co‐occurrence patterns: if some species are frequently found together, the presence of some of them at a site indicates that the site has both the biotic and abiotic conditions suitable for the missing species.	Large datasets of sampling units with records of species composition. Users can decide whether to use a training dataset or not.	For each species, it is generally based on the lowest value obtained at the site where the species is present. Outlier removal is an additional option.	Large datasets are needed. More rare species in a dataset might result in fewer robust estimates.
Species ecological preferences obtained from literature and databases (e.g., Ellenberg indicator values)	Monographs indicating species abiotic and biotic preferences (realized niche). The Ellenberg indicator values are an example for the Central European flora. Envelopes around a community mean Ellenberg values determine which species are included or excluded from the species pool.	Exhaustive monographs or databases of ecological preferences for the flora or fauna of a given region. These are built on field experience and/or results of experiments.	The size of the envelope around the community mean can vary (broader envelopes indicating larger species pools).	Large datasets with species composition data are not required, but comprehensive monographs or databases of ecological preferences are often unavailable. Important choice of ecological gradients and their weight in the calculations.
Species distribution modeling (e.g., using Biomod)	The various models of the species environmental requirements are computed based on the environmental conditions at the sites occupied by a species. The environmental conditions at a target site determine the likelihood of each species occurring there.	Large training dataset of composition data or only records of presence data (for single species) in the area. Environmental data (either field measures or GIS retrieved) for the records in the dataset. Environmental conditions at a target site.	Various techniques are used to transform the likelihood of occurrence into presence/absence data.	The type and precision of the environmental variables considered is crucial. More rare species in a dataset might result in less robust estimates.

## Methods

### Dataset

We extracted >50,000 vegetation plots (i.e., sampling units in which plant species' presences and abundances are recorded) from the Czech National Phytosociological Database (Chytrý and Rafajová 2003). This database stores samples from plant communities (vegetation plots) recorded in all types of vegetation throughout the Czech Republic. Each plot has been quality‐checked by local experts before being included in the database, to ensure consistency in species taxonomy, nomenclature and other possible sources of confusion. The set of plots used in this study is a selection from the database, which was then divided into a “training dataset,” used to calibrate the methods, and a “test dataset,” which was used to compare the different methods. The training dataset included 55,161 plots from all types of vegetation distributed throughout the country. It is based on the entire database containing more than 100,000 plots, which was geographically stratified in order to limit oversampling of some locations (Knollová et al. 2005). To do this, the database was first divided up based on the type of vegetation (phytosociological alliances or classes), which correspond to different habitats. Then plots within habitats were divided into geographic grid cells of 0.75 min of latitude and 1.25 min of longitude (~1.5 × 1.4 km). For each cell, up to three plots were selected using heterogeneity‐constrained resampling with Bray–Curtis dissimilarity as a measure of the differences in the species composition of the different plots (Lengyel et al. 2011).

**Figure 1:**
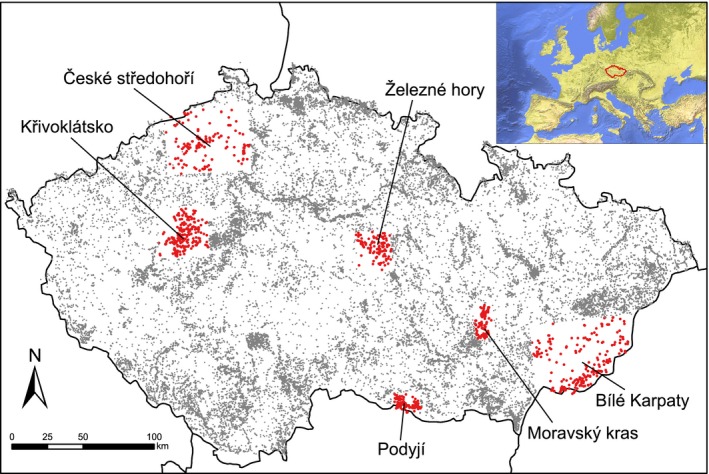
Map of the Czech Republic showing the locations of the 1083 test plots (in red) and all training plots (>50,000, in gray).

To compare the different methods, we selected 1083 plots (for which species pools were computed) as the test dataset, which includes 18 of the most abundant habitats in the whole database. These plots were from six regions of the Czech Republic (see Fig. 1 and Supporting Information). These regions were selected because all of them were comprehensively sampled and each contained most of the 18 target habitats. Within each region, a maximum of 20 plots per habitat were selected (Supporting Information). For both the training dataset and test dataset, only those samples with coordinates were used (where coordinates were given by the author of the plot, or specific geographic references recorded during the survey enabled an a‐posteriori location of the plot). For the test dataset, we only used samples for which coordinates were provided by the author. For the test plots, a further size constraint was also applied, that is, only plots with an area of 16–25 m^2^ for nonforest vegetation and 100–400 m^2^ for forest vegetation were selected, in order to standardize their size and consequently also species richness across plots.

### Species pool calculations

In the analyses, the training dataset (55,161 plots) was used to estimate the species pool for each of the 1083 test plots. For each of these 1083 plots, three types of measures were applied to compute the species pool. The resulting species pools were compared among each other and with published estimates of experts (Sádlo et al. 2007).

As in Dupré (2000), the published expert estimates (Sádlo et al. 2007) were considered to be the most accurate estimates of the species pools. Expert knowledge unfortunately is often not reproducible analytically, which limits its application to specific regions. We used the advantage of having, for the whole of the Czech Republic, expert estimates of species pools for different habitats (Sádlo et al. 2007). The expert approach was based on synthetic estimates of the affinity of plant species for broadly defined habitat types. Several criteria were combined to define this affinity. First species occurrence frequency and fidelity (according to Tichý and Chytrý 2006) to different types of habitats were computed using data from the Czech National Phytosociological Database. Second, these estimates were further complemented by consulting handbooks on the Czech flora, individual publications, and expert knowledge, particularly for less frequently occurring species (Sádlo et al. 2007).

To test the sensitivity of the comparisons between and within methods, we applied each method in different configurations (see details below). For example, each method can give a higher or lower priority for each species to be included in the species pool depending on method‐specific thresholds. For the expert approach, for example, we either included or omitted species with the lowest affinities to each species pool. We compared expert estimates against alternative, fully analytical methods. The co‐occurrence approach was based on estimates of species pools using Beals smoothing, which was proposed by Ewald (2002) and adapted by Münzbergová and Herben (2004; hereafter “Beals approach”). The Beals approach produces a probability of occurrence for a given species in each plot based on the joint occurrence of this species with other species (if a target species often occurs together with others, the presence of some of the co‐occurring species at a site is a good indicator that the target species is also likely to be in the species pool). Here, the probability of occurrence was calculated either using the 55,161 vegetation plots in the training dataset, or the 1083 plots in the test dataset. To translate such probabilities into species presences and absences in the species pools of particular communities, species‐specific thresholds were applied, as proposed by Botta‐Dukát (2012) and Münzbergová and Herben (2004), to define the species that can be included in the species pool. For each species, the threshold is the lowest Beals smoothing value for those plots in which the species is present, therefore correcting for false positives in the estimates (see later). The threshold was then applied excluding, or not, outliers as proposed by Botta‐Dukát (2012). A customized version of the function “beals” (De Cáceres and Legendre 2008), in package “vegan” (Oksanen et al. 2015), in software R (R Core Team 2014) was used.

For the approach using the species' ecological requirements, we used the method proposed by Pärtel et al. (1996), which is based on species' Ellenberg indicator values (referred to as the “Ellenberg approach”). Ellenberg values indicate the ecological preferences of species, that is, their realized niche, along different environmental gradients, mostly based on field observations (Ellenberg et al. 1992), and are available for most of the species of plants in the study area. Following Dupré (2000), first the mean and standard deviation (SD) of the Ellenberg values (for light, temperature, moisture, soil reaction, and nutrient indicator values) for the species present in each plot was computed. The plot mean was computed either considering or not the differences in species cover. As the results were very similar, only those means based on presence/absence are presented. Then, species in the training dataset were included in the species pool of that plot if their Ellenberg values for these factors were not exceeding given differences from the mean for the plot. The maximum differences allowed were 1.5, 2, or 2.5 SD units from the mean for the plot (Pärtel et al. 1996; Dupré 2000), with the distance being averaged over all five Ellenberg values. We included in the species pool of a given plot all the unrecorded species that were below these thresholds.

Species pools were finally estimated using species distribution modeling (SDM). As there are various modeling techniques, we used the modeling approach in the widely available and standardized platform Biomod (Thuiller et al. 2009), which encompasses different modeling approaches. In principle species, distribution modeling uses the environmental characteristics of the sites where the species is present (in this case, those in the training dataset) to determine the environmental conditions preferred by each species. Models were run on different random selections of the samples in the training dataset (>50,000 plots), that is, 80% of the dataset was used to calibrate the models and the remainder used for testing. Then, based on whether the environmental conditions of a given test plot are suitable for a given species, species are included or not in the species pool of a given community (plot). First, for each plot we derived a set of environmental parameters representing climatic, topographic, soil, and habitat conditions. As these variables (*n* = 37) were highly correlated with one another, we selected the subset with the lowest correlation (|*R*| < 0.6) that included a representative set of important environmental variables (Supporting Information). To calculate climatic variables, we used monthly mean, maximum, and minimum temperatures and data on precipitation provided by the Czech Hydro‐Meteorological Institute (CHMI) for grids with a 0.5‐km resolution. The grids were interpolated by regression kriging using a digital altitude model as an auxiliary predictor. From these values, we calculated biologically relevant variables using the “biovars” function in the “dismo” R package (Hijmans et al. 2013; for details, see Supporting Information).

While using Biomod, we applied several modeling techniques (GLM, GAM, Random Forests, i.e., “RF,” and Classification Trees). These produced probability values for species belonging to the species pool at a given site. Such probability values were transformed into presence/absence, that is, binary values, using several standard evaluation methods in Biomod, based on thresholds that resulted in the best predictions, evaluated as the best scores of TSS, ROC, and KAPPA (Allouche et al. 2006). It is noteworthy that these thresholds, different from those used for the Beals approach, account for both false‐positive and false‐negative predictions. Of all the possible combinations of these tools, we chose GLM and RF, because they provide predictions for the greatest number of species (1004 and 1012 predicted species, respectively). In this sense, we disregarded the option of an ensemble forecast, which combines species predictions of the different modeling techniques, because several of these techniques fail when species are only present in a few plots in the training dataset. We then chose the results obtained using TSS and KAPPA as results obtained using ROC were strongly correlated with those obtained using TSS (*R* ~ 0.98). We restricted our analyses to the final set of 1004 species present in at least 50 plots in the training dataset. This set of common species allowed meaningful comparisons of a sufficient number of species using the different methods.

We then considered other comparisons between Beals and Biomod, the two methods that provided an estimate of the probability of a species occurring in a plot. Each method uses specific thresholds of the presence/absence estimates of the probability. In order to compare the different methods, in terms of both size and composition, we need to transform the presence/absence estimates for species pools, with the risk that the choice of threshold could affect the results. While we used the thresholds best suited to each method (i.e., those that are used routinely), we also tried to use a common threshold. We then applied the one used in the Beals approach in the Biomod approach, as the correction for false positive does not seem to be an important concern in Beals approach (Münzbergová and Herben 2004).

### Comparisons of methods

To compare methods meaningfully, we compared their estimates of the portion of a species pool that is not locally present, that is, we effectively compared their estimates of dark diversity. We did it in order not to overemphasize the congruence between methods. The comparisons were based on four tests:

- An assessment of how congruent the sizes of the species pools predicted by the different methods was. For this test, we computed Pearson correlation (*R*) between the sizes of the dark diversity predicted by a pair of methods. We also used standardized major axis (Type II) regressions (Warton et al. 2006), which do not assume a unidirectional effect between variables, to verify if the slope of the relationship between variables differed from 1 and the intercept from 0.
- We assessed how much congruency was observed in the recorded species composition and dark diversity estimated by each pair of methods. This comparison was made, primarily, for a comparable range of sizes in dark diversity using the test dataset. This approach was followed to minimize the potential effect of comparing species pools varying systematically in size. In addition, the matching was expressed as overlap in composition, using the Simpson overlap coefficient (sometimes called “Szymkiewicz‐Simpson”), which is related to Sørensen compositional similarity. Using this approach, it is possible to compare matches in species composition independently of differences in species richness (Lennon et al. 2001; Baselga 2010). The coefficient specifically measures the overlap between two sets as the size of the intersection (common species) divided by the smaller of the sizes of the two sets. We then used null models with randomizations to test whether the overlap between the estimates of each of the three methods and the expert estimates was greater than expected by chance. In the randomizations (999 for each test plot), probability of species occurring in a species pool of a test plot was set equal to its frequency in the training set (i.e., the whole Czech Republic), in order to give most frequent species a greater chance of occurring in random selections. The number of species equal to the one recorded in the target species pool was randomly selected in each draw.
- We assessed how much congruency there was in detecting similar changes in composition between the dark diversities of the test plots. This involved determining whether the change in composition between species pools estimated by one method was similar to that estimated by another. To do this we computed, for each estimate of the dark diversity, 1 minus Sørensen similarity between the dark diversity of all 1083 test communities. Notice that, in contrast to test (II), where we used overlap in species composition, here we focus on total changes in species composition, which include both differences in richness and species replacements in dark diversities, in order to have a measure of total change across plots within a given method. To assess the agreement in species composition predicted by the different methods, we used the Mantel test with 999 permutations in the “vegan” package. To further validate the results of the Mantel test, a Coinertia analysis was carried out using the function “coinertia” in R package “ade4” (Dray and Dufour 2007). As the Coinertia analysis provided qualitatively, the same results as the Mantel test the results of this analysis are not shown.
- After applying the three tests described above (I–III) using the data of 1083 plots (plot‐level analyses), we also combined the plots within each type of vegetation to produce habitat estimates of dark diversity (habitat‐level analyses). In this study, the expert approach (Sádlo et al. 2007) originally provided estimates of species pool for 88 habitats (18 of them used in this study), while other methods estimated the species pool for each plot separately. Species pools of plots within habitats may differ because of between site heterogeneity. Plot‐level species pools are therefore expected to be subsets of the habitat‐level species pool. The effect of specific local factors (e.g., variations in land use, soil conditions) can be, therefore, leveled off using a habitat‐level comparison. Hence, we created habitat level estimates by pooling the plot‐level species lists and comparing them. To do this, we determined in how many plots, within each habitat, each species was present in the dark diversity (i.e., species pool of a plot excluding recorded diversity). We expressed this as a percentage of plots within a habitat. We then “transformed” these percentage values into zeros and ones by defining a species to be a part of the dark diversity of a given habitat if the species was present in the dark diversity of at least 5% of the plots from that habitat. We changed the threshold from 1% to 40% of plots and obtained results consistent with those finally presented (obviously with smaller dark diversities with greater percentages).

## Results

### Congruence in size

**Figure 2:**
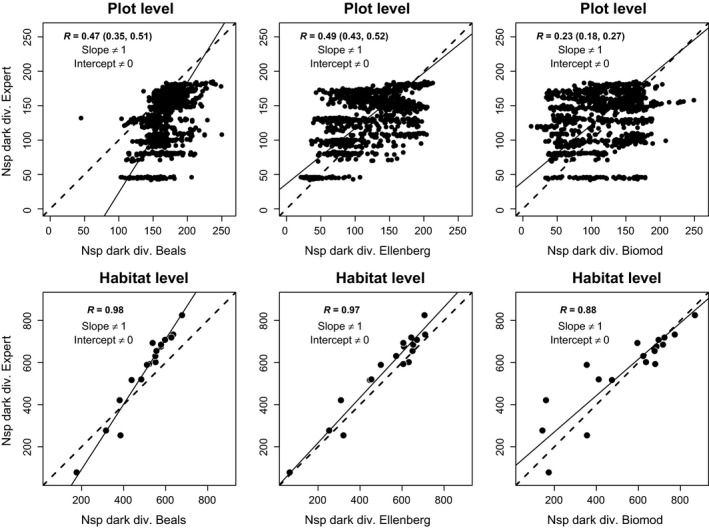
Relationship between the sizes of the dark diversity (species pool minus observed diversity) predicted by the different methods and experts. Results are for plot‐level and habitat‐level analyses (see Methods). The dashed line refers to the expected 1:1 relationship, and the solid line follows the standardized major axis regression. Each panel indicates the Pearson correlation (*R*) of the size of dark diversity for pairs of methods. The *R* value includes the points displayed in the figures for plot‐level analyses and the range of values obtained when choosing different variants using both methods. Cases in which the slope of the standardized major axis regression was different from the expected 1:1 relationship and the intercept was different from zero are indicated (see text within each panel). The figure refers to the results obtained based on expert judgment, excluding species with the lowest affinity, Ellenberg values for threshold set to ±2 SD units, Beals index estimated using the >50,000 plots as a training dataset and removing outliers, while Biomod refer to the GLM + Kappa approach. The range of *R* values at the plot level indicates the effect of the sensitivity analysis (see Table 2 for all pairwise comparisons). Nsp = number of species.

The results for all the methods, with the general exception of the species distribution modeling using Biomod, roughly converged in identifying where the dark diversity was high or low, but the different methods also provided a generally low level of agreement within plot‐level analyses (*R* ~ 0.4; Fig. 2 and Table 2; all correlations were significant, *P *<* *0.001). Convergence increased considerably when the estimates were compared at the habitat level (Fig. 2). Using different thresholds in the different methods affected the average size of the dark diversity. For this reason, while presenting the different results, we also focused on the results with a similar average size and range of dark diversities across methods (points displayed in Fig. 2; other results are shown in Table 2). At the plot level, both co‐occurrence and species ecological requirements (Beals and Ellenberg approaches, respectively) provided estimates most comparable with those of experts, with the Ellenberg approach giving slightly better correlation on average and a slope closer to 1. In both cases, however, standardized major axis regressions detected a non 1:1 relationship (the slope was significantly different from 1). At the habitat level, Ellenberg and Beals approaches both provided estimates that were very strongly correlated with those of the experts (Fig. 2), but again the Ellenberg approach generally giving a better correlation with a better slope (not different from 1) and intercept (not different from 0). Compared to the expert approach, the Beals approach generally produced larger species pools (paired t‐test, *P *<* *0.05), particularly at locations with smaller dark diversities (*n* = 1083 for all tests including those mentioned below). For the same conditions, but only at the plot level, the Ellenberg approach also produced slightly smaller species pools.

**Table 2:** Correlations between estimates of dark diversities at the plot level using different methods and different thresholds

	Expert		Beals			Ellenberg			Biomod			
	All	With affinity	TrainingNoOut	TrainingOut	NotrainingNoOut	1.5SD	2SD	2.5SD	GLM & TSS	GLM & KAPPA	RF & TSS	RF & KAPPA
Expert												
All	–	–	–	–	–	–	–	–	–	–	–	–
With affinity	0.91	–	–	–	–	–	–	–	–	–	–	–
Beals												
TrainingNoOut	0.52	0.47	–	–	–	–	–	–	–	–	–	–
TrainingOut	0.41	0.37	0.93	–	–	–	–	–	–	–	–	–
NotrainingNoOut	0.36	0.35	0.52	0.54	–	–	–	–	–	–	–	–
Ellenberg												
1.5SD	0.44	0.43	0.43	0.41	0.29	–	–	–	–	–	–	–
2SD	0.48	0.46	0.48	0.44	0.32	0.96	–	–	–	–	–	–
2.5SD	0.52	0.49	0.50	0.48	0.33	0.93	0.96	–	–	–	–	–
Biomod												
GLM & TSS	0.28	0.27	0.19	0.16	0.15	0.10	0.12	0.20	–	–	–	–
GLM & KAPPA	0.24	0.24	0.25	0.22	0.18	0.10	0.12	0.20	0.47	–	–	–
RF & TSS	0.23	0.23	0.30	0.27	0.25	0.08	0.09	0.12	0.10	0.65	–	–
RF & KAPPA	0.20	0.19	0.29	0.28	0.25	0.09	0.09	0.13	0.08	0.65	0.99	–

Approach	Method	Meaning
Expert	All	All species predicted by Sádlo et al. (2007) are included, irrespectively of their affinity for a given species pool
	With affinity	All species predicted by Sadlo et al. (2007) are included, excluding those with lower affinity for a given species pool
Beals	TrainingNoOut	Using training dataset (>50,000 samples) and using the approach by Botta‐Dukát (2012) which removes outliers and apply randomization to compute thresholds
	TrainingOut	Using training dataset (>50,000 samples) and using the approach by Münzbergová and Herben (2004) to compute thresholds
	NotrainingNoOut	Not using a training dataset (only the ~1000 target communities are considered) and using the approach by Botta‐Dukát (2012) which removes outliers and apply randomization to compute thresholds
Ellenberg	1.5SD	Using mean and 1.5 standard deviation units around the community mean as an envelop
	2SD	Using mean and 2 standard deviation units around the community mean as an envelop
	2.5SD	Using mean and 2 standard deviation units around the community mean as an envelop
Biomod	GLM & TSS	Using GLM as modeling tool and TSS to define the threshold
	GLM & KAPPA	Using GLM as modeling tool and KAPPA to define the threshold
	RF & TSS	Using Random Forest as modeling tool and TSS to define the threshold
	RF & KAPPA	Using Random Forest as modeling tool and KAPPA to define the threshold

For each method, as mentioned above, the dark diversity at a site depended on the type and strength of the thresholds used. Not unexpectedly, the less constraining the threshold, the greater the dark diversity. Less constrained thresholds generally also resulted in stronger correlations with other methods, particularly at the plot level, as bigger pools generally imply more species are shared. For example, when the threshold for the expert method was not applied (i.e., not removing the species with the lowest habitat affinity, which are often rare species, whose ecological optimum is in another habitat), the dark diversity at the plot level was on average two times greater. Including species with the lowest habitat affinity strengthened the correlation between the dark diversities of experts and those provided by all the other methods (generally produced the higher values in the correlation ranges in Fig. 2, see Table 2). Changing thresholds in the Ellenberg approach, from 1.5 to 2 SD and from 2 to 2.5 SD produced increases in average dark diversity of 15% and 22%, respectively. For Biomod, random forest generally resulted larger dark diversities (75% larger for the KAPPA threshold and 48% for TSS threshold). For the Beals index, a larger dark diversity was obtained when >50,000 plots of the training dataset were compared and the outliers were not removed (removing outliers decreased species pool size on average by 16% and using only 1083 plots decreased it by 38%). Generally, not using a training dataset in the Beals approach produced poorer results (lower *R*) compared to other methods. When the results of the Beals and Biomod approaches were compared using the same threshold (only accounting for false negatives), the dark diversity estimated using Biomod was twice that estimated by Beals, but did not increase the correlations between them and those of the Biomod and expert approaches, and therefore, this was not further explored.

### Congruence in species composition

**Figure 3:**
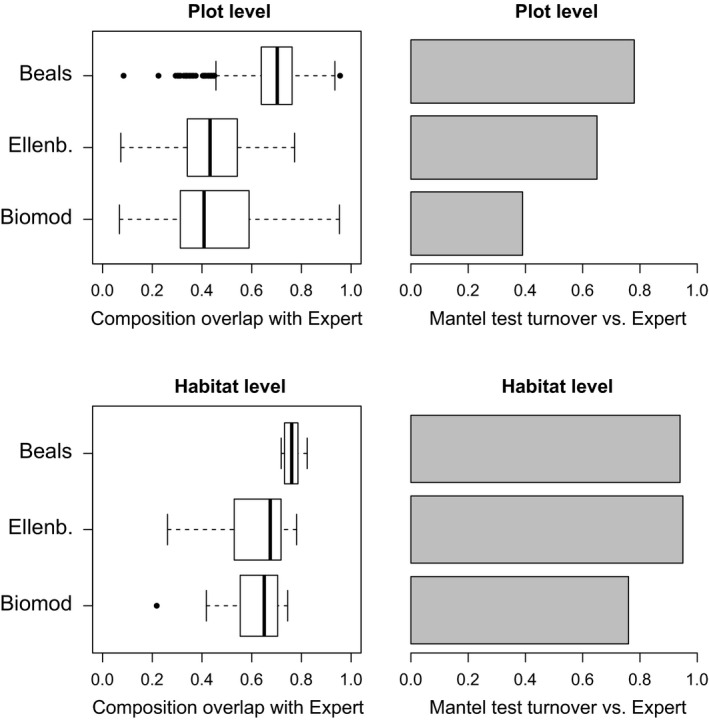
Left panels: agreement in species composition between the species pools estimated for pairs of methods, as in Fig. 2. Results are shown for both plot‐level and habitat‐level analyses (see Methods). The agreement is expressed in terms of the overlap (Simpson coefficient) between the estimates for the species pool of a given test community predicted by the different methods (i.e., comparison between methods for a given plot; see Methods). For simplicity, the expert approach is considered as a benchmark. Right panels: a Mantel test (*R* statistic) of the changes in species composition recorded across the species pools of the 1083 plots using different methods (turnover between pairs of species pools estimated using Bray–Curtis dissimilarity between pairs of plots within a given method). Exp, expert estimates; Ell, Ellenberg approach; Biom, Biomod approach; Beals, Beals approach. In both figures, the species recorded in a plot were removed in order not to overemphasize congruence in predictions of the different methods.

The comparison of species composition was made, primarily, using for each method the configuration that produced a comparable range of dark diversities across the test dataset (Fig. 2). This was done to further minimize, as mentioned above, the effect of comparing dark diversities that vary systematically in size. The test based on composition overlap (Fig. 3, left panels) showed a reasonably good agreement in terms of the species detected by a given analytical method and the expert estimates. This was particularly true using the Beals approach, which provided significantly better results than the other methods when compared to the expert evaluation at the plot level (in both cases paired t‐test provided *P *<* *0.001; mean overlap = 0.70, SD = 0.11). In general, at the plot level, there was an overlap of slightly less than half of the species estimated using the Ellenberg and Biomod methods and expert estimates (for Ellenberg mean = 0.44 and SD = 0.13, for Biomod, mean = 0.45, SD = 0.17). Using the Beals method, 94% of the test plots showed an overlap with the expert estimates, which was significantly greater (*P *<* *0.05) than expected by chance (two‐tailed test). Using the Ellenberg and Biomod estimates, only ~45% showed a greater matching than expected by chance (50% and 43%, respectively). At the habitat level, the agreement with expert estimates increased for all methods (paired t‐test *P *<* *0.001 between habitat‐ and plot‐level analyses), with all having a mean overlap >0.6. For all methods, and for each habitat, the match was significantly greater (*P *<* *0.05) than expected by chance (two‐tailed test).

### Congruence in the turnover in species composition

The extents of the overlaps in the species compositions of the dark diversities of the different sites (Fig. 3, right panels) predicted by the different methods was generally comparable, particularly at the habitat level. Overall, Beals and Ellenberg approaches predicted species compositions changes closest to the expert estimates (Mantel *R* ~ 0.7 at the plot level and *R* ~ 0.9 at the habitat level), with all values significantly greater than expected by chance. The Beals approach provided the strongest agreement at the plot level. As for the agreement in terms of dark diversity, looser thresholds increased the size of the species pools and resulted in fewer changes in the species composition of the dark diversity of the communities considered (not shown), because more species were shared. Again, in order to carry out a comparable test, we focused on dark diversities composed of a similar number of species. The test indicates that the species composition changes in the dark diversity predicted by most of the different methods is similar. In other words, this test shows that there is general agreement regarding the prediction of the distribution of dark diversity in a region.

## Discussion

The aim of this study was to assess how different methods can be used to estimate habitat‐specific species pools, that is, the set of species that could potentially live in a particular site, especially those species not already recorded by sampling. The set of species that can inhabit a particular habitat, its specific species pool, but not currently recorded in that habitat, is called “dark diversity” (Pärtel et al. 2011; Zobel 2016). We selected methods that can be used to estimate the size and composition of dark diversity and use similar techniques (Pärtel et al. 1996; Dupré 2000; Ewald 2002; Thuiller et al. 2009). There is some degree of agreement between the results of the different methods and the expert estimates available for the region studied (Sádlo et al. 2007). Although a convergence of results was apparent at the habitat level, there were however substantial differences in the different measures, in terms of the sizes and species compositions of dark diversity, at the plot level.

The similarity in the dark diversity estimates at the habitat level is encouraging in terms of developing practical tools for defining habitat‐specific species pools in a region. The expert approach considered (Sádlo et al. 2007) was based on estimates of species pools for different types of habitats (88 types originally considered, 18 of which were well enough represented in our dataset). All other approaches provided separate estimates for each plot. The high similarity recorded at the habitat level partially reflects the scale used in the expert approach. However, the results of all the methods converged at the habitat level. This indicates that dark diversity of single plots within a habitat can differ because of potential differences in specific local factors, for example, land use and soil conditions, causing within‐habitat heterogeneity. By pooling plot‐level estimates of dark diversity, the effect of within‐habitat heterogeneity (not present in the expert estimates) is reduced and the convergence of the results of the different methods increased. This convergence in dark diversity estimates at the habitat level makes it possible to more accurately estimate which species in a particular habitat are likely to be part of its species pool and dark diversity. This positive result has a clear implication for theoretical and applied studies using the species pool concept, for example, comparing species richness along gradients and in different habitats, understanding the mechanisms regulating the biodiversity in assemblages from habitat‐specific species pools, and assessing dispersal limitation and extinction risk for nature conservation, restoration, and management of invasive species (de Bello et al. 2012; Carstensen et al. 2013; Cornell and Harrison 2014; Lessard et al. 2016; Zobel 2016). For these purposes, defining habitat‐level dark diversity is essential and this study shows that the existing quantitative methods can be used even when there are no expert estimates.

Differences between methods, mainly at the plot level, are not surprising (Dupré 2000) particularly given the different approaches of the different methods, each of which is based on different assumptions and uses different types of data (Table 1). These differences between the methods highlight the different nature of each approach, the type and quality of data used and a number of methodological choices inherent to each method, particularly to account for specific local conditions. Among the different methods, the Beals smoothing (co‐occurrence approach) produced the results that matched the estimate of local experts most closely at the plot level. This confirms the results of a completely different approach that of Lewis et al. (2016b), who accurately estimated the additional species found around sampled plots using Beals approach. In our study, Beals approach was slightly inferior to that based on species ecological requirements using the Ellenberg approach (see next paragraph for details) for determining the size of the species pool, compared to expert estimates, but was better in detecting the match in species composition and changes in composition between sites. The co‐occurrence approach is relatively easy to apply using existing algorithms, although it needs a comprehensive and well‐stratified datasets of the species compositions of different types of habitat, which may not be always available. Existing continental and global initiatives to collect biodiversity data will eventually provide sufficient data to apply this method. We found that using a training dataset, or in general a large set of samples, can improve the reliability of the results at the plot level (e.g., Table 2). There was also a tendency for the co‐occurrence approach to yield the most distinctive change in slope when comparing the size of the species pool to that estimated by experts and other methods (Fig. 2). Thus, comparing the size of the local diversity with the species pool (Dupré 2000; Lepš 2001) using this approach should be done with care.

The approach based on the ecological preferences of species, in this case using Ellenberg indicator values available for Central European vascular species of plants, is an alternative to the co‐occurrence approach, particularly when comparing recorded diversity with species pool size. Both methods provide estimates of the realized niches of species, which reflect both the biotic and abiotic conditions in which species can occur. Contrary to the co‐occurrence approach, the ecological preference method does not require large datasets of species composition but only evaluations that characterize the preference of species for different habitats. Monographs containing species indicator values are available for a number of European countries (for review see, e.g., Diekmann 2003), and these can be useful for generating species pools of a size that is similar to that estimated by local experts (both at the plot and habitat levels). Yet more information is required for several regions of the World and for several different types of organisms. Our results generally indicate that for detecting species composition at a site the Ellenberg approach is less accurate than expert estimates. In this sense, the results generally agree with the findings of Dupré (2000), who compared the Ellenberg approach with expert estimates, and those of Lewis et al. (2016b), who compared the estimates obtained using the Ellenberg approach with those based on recording species in sample plots. In addition, we expect that information on species preferences along more than five environmental gradients, as used here, will further improve the predictions by these methods at the plot level. Particularly, information on species tolerance to disturbance or extreme events are likely to further increase the precision of these methods in estimating the effect of specific local conditions.

The species distribution modeling generally provided a lower level of agreement with the other methods, particularly at the plot level, probably because at this scale, fine‐scale environmental information in terms of type of variable and their specific spatial resolution are required. Although we included CORINE land cover (which includes some information on broadly defined habitat types) and other information as predictors, precise information on variations in land use (grazing, mowing, fertilization, and logging) or local soil and microclimatic variables were not available. Land‐use practice, for example, can determine the species potentially available to colonize a site, but this information is often not available in many datasets (de Bello et al. 2010). As such, species distribution models are likely to be less robust than other methods at the scale of biological communities, but useful at the habitat scale, where the coarseness of environmental variables matches the aggregated species data. In general, at coarser resolutions, for example, working with data on grids greater than 1 × 1 km, the effect of particular local land‐use effects should decrease and the match between resolution of environmental variables and species data should increase, making estimates based on general environmental variables more reliable. While we excluded a priori plots from the training dataset with indefinable coordinates, we cannot exclude that, on some occasions, the low precision of the plot coordinates in the training dataset could have partially weakened the results of species distribution models. It should also be noted that, among the methods considered the variants applied within the SDM approach gave the results that were least correlated with each other (Table 2). In this sense, the particular parameters used can greatly affect the results. This is not surprising (Elith and Leathwick 2009) and the problem can be partly solved using combinations of different methods (Thuiller et al. 2009). However, ensemble forecasts are only suitable for widespread species for which there is a lot of data or for large regions, so that the number of low‐frequency species is minimized.

The relatively little similarity in the results obtained using the three analytical approaches and those of local experts' at the plot level indicates it is currently difficult to accurately estimate the size and species composition of the species pool of target communities. While expert knowledge cannot always be a priori seen as the best method for estimating species pools, it is generally the most holistic approach (Dupré 2000; Sádlo et al. 2007). In regions where expert knowledge is not available, it is likely that developments in methodology and more data could further improve the accuracy of the estimates of the size and composition of species pool for given plots. It is noteworthy that the estimates of species pools were consistent in detecting compositional changes across sites, and, therefore, in distinguishing the species pools of different sites (Fig. 3 right panels). This indicates that the existing tools can be more safely used to define the set of species that are unlikely to occur in a given habitat. This is an encouraging message as it does indicate it is possible to improve the evaluation of the role of regional processes in ecological communities, particularly if we work in a system for which there is little background information.

Our results generally show that no analytical method provides a priori, and in all conditions, better estimates of species pools than any other method, although the co‐occurrence approach can be used if sufficient data is available and no expert estimates are available. The different methods are all theoretically valid and might be preferred in different situations depending on the type and quality of data available. Our study highlights the potential and present limitations to estimating species pools. It is therefore necessary to stress that the choice of the methods used to estimate the species pool at a site need to be carefully considered. More effort is needed to improve the definition of the species composition and size of a particular species pool, both for the evaluation of local processes in communities and for a more rigorous approach in biodiversity conservation. An important methodological choice is the threshold used in each model to decide whether a species in a region is included in the species pool. The likelihood of species occurrence, as recently suggested, could be directly used to define the size of species pools (Karger et al. 2016). At the same time, as shown here, it might be possible to use models to make predictions using several thresholds (Lessard et al. 2016). Based on the differences in the estimates, it should be possible to derive an estimate of uncertainty in the measures of species pools, which would provide a measure of the confidence that can be placed on the biological conclusions reached. It is possible to decide on several thresholds for each method, and their definition will also depend on the purpose for which the species pools are computed. For example, it might be better to use more inclusive methods in conservation, as it is less harmful to include species that are unlikely to occur in the target community than to exclude those species that could potentially occur there. In this sense the use of several thresholds could provide less subjective estimates and more robust predictions that take account of the uncertainty of the methods. We conclude that analytical methods may well mimic holistic estimates of species pools. However, the different methods have their own strengths and weaknesses.

## Conflict of Interest

None declared.

## Supporting information


**Table S1**. Locations of the six areas for the test communities and the representation of the vegetation types considered.
**Table S2**. Variables used in the species distribution models with Biomod.
