# Supplementary material for: Measuring size and composition of species pools: a comparison of dark diversity estimates
Source: Ecol Evol. 2016 May 20;6(12):4088–101. doi: 10.1002/ece3.2169 (PMC4877358; doi:10.1002/ece3.2169)
Supplement: Supplementary file 1 — Table S1. Locations of the six areas for the test communities and the representation of the vegetation types considered. Table S2. Variables used in the species distribution models with Biomod. [file ECE3-6-4088-s001.docx]

Table 1. Locations of the six areas for the test communities and the representation of the vegetation types considered.

Table 2. Variables used in the species distribution models with Biomod. Monthly climatic data were provided by the Czech Hydrometeorological Institute (CHMI) in grid with 0.5 km resolution. For vegetation plots sampled before 1985, we used monthly mean data from the period 1960-1985, for vegetation plots sampled after 1985, we used monthly mean data from the period 1986-2010.

| Variable | Resolution | Calculation | Data source | Reference |
| --- | --- | --- | --- | --- |
| ***Climate*** |  |  |  |  |
| Potential evapotranspiration | 0.5 km | modified Thorntwaite method | CHMI | Wilmott et al. (1985) |
| Mean diurnal temperature range | 0.5 km | mean difference of monthly maximum and minimum temperatures | CHMI | Hijmans et al. (2013) |
| Mean temperature of the driest quarter | 0.5 km |  | CHMI | Hijmans et al. (2013) |
| ***Topography*** |  |  |  |  |
| Elevation | 90 m |  | SRTM DEM | Jarvis et al. (2004) |
| Slope | 90 m | Horn method | SRTM DEM | Horn (1981) |
| Annual solar radiation | 90 m | Solar Analyst in ArcGIS | SRTM DEM | Fu and Rich (2000) |
| ***Soil*** |  |  |  |  |
| Soil pH | 1 km | topsoil pH | Harmonized World Soil Database | FAO/IIASA/ISRIC/ISS-CAS/JRC (2012) |
| ***Habitat*** |  |  |  |  |
| Enhanced Vegetation Index | 1 km | mean of the monthly MODIS EVI data (2011-2012) | WorldGrids.org | Reuter and Hengl (2012) |
| CORINE landcover | 250 m | version 2000 | European Environment Agency | Bossard et al. (2000) |

References

Bossard M., Feranec J. and J. Otahel. 2000 CORINE land cover technical guide: addendum 2000. European Environment Agency, Copenhagen.

FAO/IIASA/ISRIC/ISS-CAS/JRC. 2012. Harmonized World Soil Database (version 1.2). Rome: FAO.

Fu, P. and P. M. Rich. 1999. Design and implementation of the Solar Analyst: an ArcView extension for modeling solar radiation at landscape scales. Proceedings of the 19th Annual ESRI User Conference, San Diego, USA.

Hijmans, R. J, Phillips, S., Leathwick J. and J Elith. 2013. Dismo: Species distribution modeling. R package version 0.9-3. <http://CRAN.R-project.org/package=dismo>

Horn, B. K. P. 1981. Hill shading and the reflectance map. Proceedings of the IEEE 69: 14–47.

Jarvis, A., Reuter, H. I., Nelson, A., and E. Guevara. 2008. Hole-filled SRTM for the globe. Version 4. (<http://srtm.csi.cgiar.org>).

Reuter, H.I. and T. Hengl. 2012. Worldgrids—a public repository of global soil covariates. In: Digital Soil Assessments and Beyond, Sydney, Australia, CRC Press.

Willmott, C., Rowe, C. and Y. Mintz. 1985. Climatology of the terrestrial seasonal water cycle.
